# Identification of Novel Inhibitors of *Escherichia coli* DNA Ligase (LigA)

**DOI:** 10.3390/molecules26092508

**Published:** 2021-04-25

**Authors:** Arqam Alomari, Robert Gowland, Callum Southwood, Jak Barrow, Zoe Bentley, Jashel Calvin-Nelson, Alice Kaminski, Matthew LeFevre, Anastasia J. Callaghan, Helen A. Vincent, Darren M. Gowers

**Affiliations:** 1 School of Biological Sciences and Institute of Biological and Biomedical Sciences, University of Portsmouth, Portsmouth PO1 2DY, UK or arqam.alomari@uomosul.edu.iq (A.A.); robert.gowland@port.ac.uk (R.G.); callum.southwood@myport.ac.uk (C.S.); jak.barrow@myport.ac.uk (J.B.); zoe.bentley2@myport.ac.uk (Z.B.); jashel.calvinnelson3@myport.ac.uk (J.C.-N.); alice.kaminski4@myport.ac.uk (A.K.); matthew.lefevre4@myport.ac.uk (M.L.); anastasia.callaghan@port.ac.uk (A.J.C.); helen.vincent@port.ac.uk (H.A.V.); 2 Department of Basic Sciences, College of Agriculture and Forestry, University of Mosul, Mosul 41002, Iraq

**Keywords:** antibacterial target, chemical inhibitors, DNA ligase, molecular docking, NAD^+^-dependent

## Abstract

Present in all organisms, DNA ligases catalyse the formation of a phosphodiester bond between a 3′ hydroxyl and a 5′ phosphate, a reaction that is essential for maintaining genome integrity during replication and repair. Eubacterial DNA ligases use NAD^+^ as a cofactor and possess low sequence and structural homology relative to eukaryotic DNA ligases which use ATP as a cofactor. These key differences enable specific targeting of bacterial DNA ligases as an antibacterial strategy. In this study, four small molecule accessible sites within functionally important regions of *Escherichia coli* ligase (EC-LigA) were identified using *in silico* methods. Molecular docking was then used to screen for small molecules predicted to bind to these sites. Eight candidate inhibitors were then screened for inhibitory activity in an *in vitro* ligase assay. Five of these (geneticin, chlorhexidine, glutathione (reduced), imidazolidinyl urea and 2-(aminomethyl)imidazole) showed dose-dependent inhibition of EC-LigA with half maximal inhibitory concentrations (IC_50_) in the micromolar to millimolar range (11–2600 µM). Two (geneticin and chlorhexidine) were predicted to bind to a region of EC-LigA that has not been directly investigated previously, raising the possibility that there may be amino acids within this region that are important for EC-LigA activity or that the function of essential residues proximal to this region are impacted by inhibitor interactions with this region. We anticipate that the identified small molecule binding sites and inhibitors could be pursued as part of an antibacterial strategy targeting bacterial DNA ligases.

## 1. Introduction

With the increasing incidence of multidrug-resistant bacterial strains [1,2], new bacterial enzyme targets are an urgent priority for disease research. DNA ligases are essential cellular enzymes. They are found across the three domains of life and play a critical role in cell survival by maintaining genomic integrity during replication, recombination and repair [3,4,5,6]. Deletion or inactivation of ligase genes results in nonviability and cell death [7,8,9]. All DNA ligases can repair single-stranded breaks (nicks) that are characterised by an adjacent 5′ phosphate and 3′ hydroxyl group, along with an intact cognate strand. As part of the nucleotidyl-transferase superfamily of enzymes [10], DNA ligases catalyse a three-step reaction mechanism that involves the stepwise covalent transfer of a single AMP group to drive phosphodiester bond reformation at a nick. AMP is initially transferred from a metabolic ATP or NAD^+^ cofactor to a lysine residue in a KxDG motif (where x can be any amino acid) at the ligase active site, forming an enzyme-adenylate intermediate and releasing pyrophosphate (from ATP) or nicotinamide mononucleotide (NMN) (from NAD^+^) (step 1). Upon nick location and binding by the ligase, the AMP is then transferred from the lysine to the 5′ phosphate group of the nick, forming an unstable nicked DNA-adenylate (step 2). Rapid in-line attack by the 3′ hydroxyl group on the nicked DNA-adenylate leads to phosphodiester bond formation, to restore the DNA backbone, and release of the AMP (step 3).

**Figure 1:**
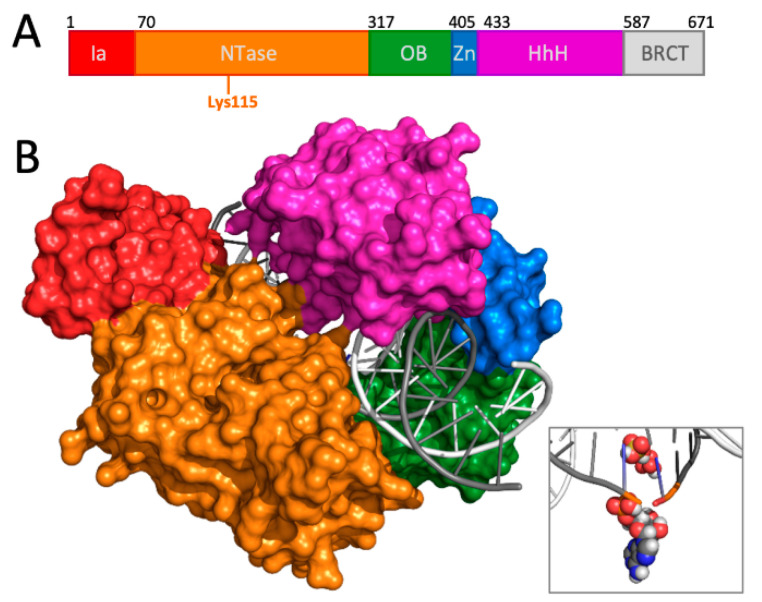
*E. coli* DNA Ligase A. (**A**) A schematic of the domain architecture of EC-LigA. From N- to C-termini (left to right) these are: Ia (N-terminal domain), NTase (nucleotidyltransferase), OB (oligomer binding OB-fold), Zn (zinc finger), HhH (helix-hairpin-helix) and BRCT (BRCA1-like C-terminus). The starting residue number of each domain is indicated above and the approximate location of the essential AMP-transfer residue (lysine 115) is indicated below. (**B**) Structure of EC-LigA (surface view) bound to a 26 bp nicked DNA-adenylate (cartoon) (PDB 2OWO [16]), with domains coloured as in Panel A. The BRCT domain was not visible in the crystal structure. The inset shows a close-up of the nick site, with the 5′-phosphoryl-AMP group (spheres: grey, carbon; red, oxygen; blue, nitrogen; white, hydrogen) on the DNA poised for nick-closure.

The metabolic source of AMP distinguishes DNA ligases into two families: all eukaryotic and archaeal ligases use ATP as the cofactor, whereas all eubacteria (and some eukaryotic DNA viruses) use NAD^+^ [4]. This functional difference, along with low sequence and structural homology between ATP and NAD^+^-utilising ligases [11], suggested that bacterial (NAD^+^) ligases could be broad-spectrum antimicrobial targets [12,13,14]. All known bacteria encode at least one highly conserved NAD^+^-dependent DNA ligase (termed LigA). LigA enzymes are highly conserved monomeric enzymes with an average chain length of 656–837 residues [5,15]. *Escherichia coli* LigA (EC-LigA), the prototype bacterial ligase is 671 amino acids long. NAD^+^ ligases, including EC-LigA, contain six functional domains (Ia, nucleotidyltransferase (NTase), oligomer binding OB-fold (OB), zinc finger (Zn), helix-hairpin-helix (HhH) and BRCA1-like C-terminal (BRCT)) (Figure 1A). The N-terminal Ia domain is unique to NAD^+^ ligases and is required for NAD^+^ binding and formation of the enzyme-adenylate intermediate [16]. The NTase domain, containing the KxDG motif, and the OB domain form the catalytic core of LigA [16], and the HhH, Zn finger and BRCT domains are involved in substrate recognition and providing the structural scaffold needed for catalysis [16,17,18]. The crystal structure of EC-LigA, in complex with a nicked DNA-adenylate, is shown in Figure 1B (PDB ID: 2OWO, [16]). This shows that the enzyme forms a clamp structure that encircles the DNA substrate. The NTase and OB-fold domains bind and orient the nick site for catalysis, the HhH domain both binds distal regions of the DNA and interacts with the NTase domain to stabilise the clamp structure, and the Zn finger domain bridges the OB-fold and HhH domains [16].

Lead generation is one of the biggest challenges in antibacterial drug discovery [19,20]. Efforts to identify inhibitors of LigA have focused on the NAD^+^ binding Ia domain that is unique to NAD^+^ ligases, and NAD^+^ analogues, in an attempt to block NAD^+^ binding and formation of the enzyme-adenylate (step 1). *In silico* prediction [21] or high-throughput screening (HTS) [12,14,22,23,24,25,26,27,28,29,30,31] approaches have identified novel LigA inhibitors including heterocyclic arylamino compounds such as chloroquine and quinacrine [22,23] tricyclic pyridochromanones [12], glycosyl ureide or amine derivatives [24]; tetracyclic indoles [25], pyridines [26] and a wide range of modified NAD^+^ and AMP derivatives [14,27,28,29,30,31]. The polycyclic nature of most of these compounds are thought to mimic NAD^+^ or AMP.

In this study, we aimed to investigate the potential of targeting EC-LigA more broadly as an inhibitory/antibacterial strategy. We applied a structure-based *in silico* screening and *in vitro* validation strategy, which we have used previously to rapidly identify inhibitory lead compounds of antibacterial targets [32,33], to identify new inhibitors that target NAD^+^ ligases. The strategy involved first identifying potential inhibitor binding sites within an NAD^+^ ligase *in silico.* We chose EC-LigA as a co-crystal structure of a nicked DNA-adenylate bound to *E. coli* LigA was available [16] and the enzyme has been well characterised by detailed mutational-mapping performed by the Shuman group (Table S1) [34,35,36,37,38]. We selected four sites within the EC-LigA structure that are accessible to small molecules and a chemical library of 800 small molecules was docked into each of these sites *in silico.* Eight small molecules, two predicted to bind at each of the four sites, were then screened for ligase inhibitor activity in an *in vitro* ligase assay. Of these eight small molecules, five were identified that demonstrated dose-dependent inhibition of EC-LigA activity. These five molecules are collectively predicted to target three of the four small molecule accessible sites identified. One of these sites has not been directly identified before in functional, mutational or structural studies, as being of importance for ligase activity. We anticipate that the identified small molecule accessible sites within LigA could be targeted in future screens for LigA inhibitors. In addition, the inhibitory small molecules identified could be taken forward either as a basis for the development of antibacterials targeting bacterial ligases or as molecular tools for studying ligases. 

## 2. Results

Our overall strategy to identify novel inhibitors of LigA involved three linked stages: the first stage was to identify small molecule accessible sites within functionally important regions of EC-LigA *in silico* that could be targeted by inhibitors; the second stage was to dock a small molecule library into each of these sites in order to identify small molecules that have the potential to bind to these sites and possibly inhibit EC-LigA; and the third stage was to validate the inhibitory activity of any small molecules predicted to bind to the target sites in an *in vitro* ligase assay. 

### 2.1. Identification of Potential Inhibitor Target Sites within EC-LigA

**Figure 2:**
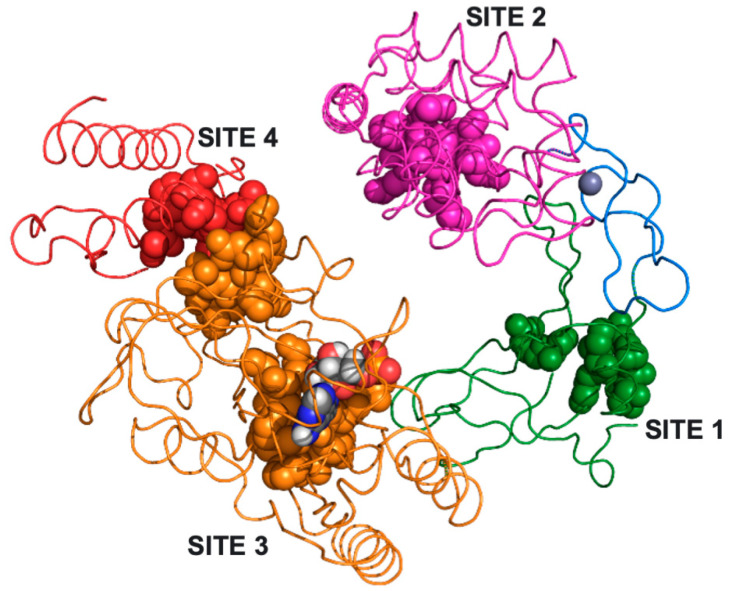
Potential inhibitor target sites in EC-LigA. Structure of EC-LigA (wire) coloured by domain (Ia, red; NTase, orange; OB-fold, green; Zn finger, blue; HhH, magenta) (PDB 2OWO [16] with the nicked DNA removed). The amino acids forming small molecule accessible sites (spheres, coloured according to domain location) were identified using the MOE site-finder tool. The sites are labelled SITE 1 to SITE 4. The AMP from the nicked DNA-adenylate (shown as spheres: grey, carbon; red, oxygen; blue, nitrogen; white, hydrogen) binds in the AMP-binding pocket within SITE 3. A Zn^2+^ ion (purple sphere) is coordinated by the Zn finger domain.

In order to identify small molecule accessible sites within EC-LigA that could be targeted by inhibitors, we first prepared an apo-EC-LigA structure by removing the nicked DNA-adenylate from the 2OWO crystal structure in the molecular docking software Molecular Operating Environment (MOE, [39]). The MOE site-finder tool was then used to identify solvent-exposed sites with a volume >5 Å^3^ that could be accessible to small molecules. A total of 32 sites (ranging in volume from 8 to 74 Å^3^) were found. Four sites were selected as potential inhibitor target sites (Figure 2; Tables S2–S5).

The selected sites were evaluated for their potential functional significance by comparing their amino acid composition and location within the EC-LigA structure to a series of mutational-mapping studies conducted by the Shuman group [34,35,36,37,38] (Figure S1; Tables S2–S5). Site 1, the smallest but most accessible site, is located within the OB-fold domain. This domain forms part of the clamp that encircles the nick site of a DNA substrate and contains essential amino acids R333 (DNA binding), R379 (stabilises the OB-fold), and V383 and I384 (distort the DNA at the nick site) [16,37]. Although Site 1 does not contain any of the known key amino acids, we reasoned that disruption of this domain with a small molecule could affect formation of the clamp structure required for activity, indirectly affecting the function of essential residues of the clamp. Site 2 is exposed on the external face of the enzyme within the HhH DNA binding domain. Six amino acid residues within this domain have been identified as performing an essential role for EC-LigA activity: R446 (HhH-NTase domain interaction) and G455, R487, G489, G521 and G553 (DNA binding) [16,37]. Site 2 contains the essential amino acid R487 and is proximal to essential amino acids G455 and G489 and therefore small molecule binding at this site has the potential to inhibit DNA binding. Site 3 is a large internal pocket within the NTase domain that overlaps with the AMP binding pocket at the NTase active site [16,34,36,38]. It is proximal to K115, which is adenylated to form the enzyme-adenylate, and contains L116, D117 and G118 of the KxDG motif [16,34]. Essential amino acids E173 (NAD^+^ binding) and R200 (DNA binding) [16,34,36,38] are also present within this site. Blocking this site with a small molecule would most likely prevent formation of the enzyme-adenylate and subsequent steps of the ligation reaction. Finally, Site 4, the largest but least accessible site, spans regions of the Ia and NTase domains. It contains two amino acids within the Ia domain, Y35 and D36, that are essential for NAD^+^ binding and formation of the enzyme-adenylate [16,35]. Binding of a small molecule at this site could also potentially affect the interaction of the enzyme with the NAD^+^ cofactor and inhibit EC-LigA at the first step of the reaction. 

### 2.2. In Silico Molecular Docking of Small Molecules into the Potential Inhibitor Target Sites within EC-LigA

**Figure 3:**
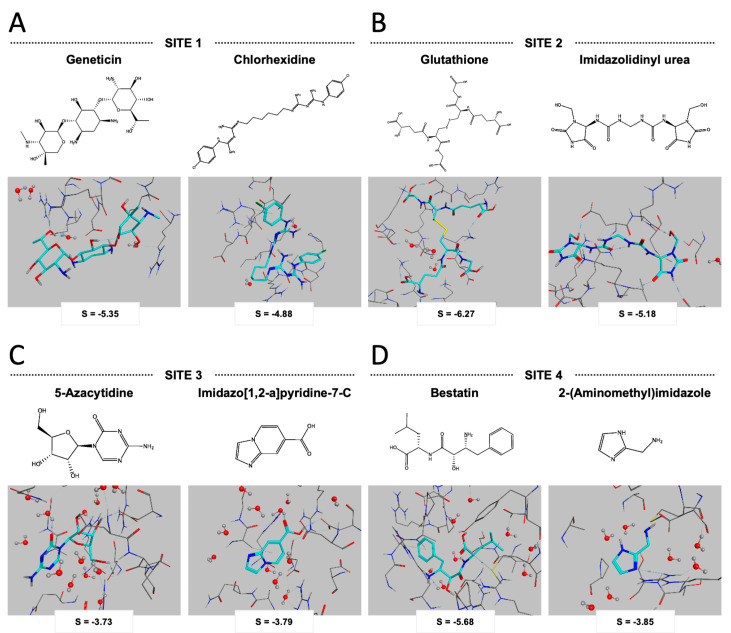
Molecular docking of small molecules into the potential inhibitor target sites of EC-LigA. The lowest energy EC-LigA-small molecule complexes for (**A**) geneticin and chlorhexidine docked into Site 1 (**B**) glutathione and imidazolidinyl urea docked into Site 2 (**C**) 5-azacytidine and imidazo[1,2-a]pyridine-7-C docked into Site 3 and (**D**) bestatin and 2-(aminomethyl)imidazole docked into Site 4. Small molecules are shown as cyan sticks with nitrogen coloured blue and oxygen coloured red. Amino acids in the potential inhibitor target site are shown as grey sticks with nitrogen coloured blue and oxygen coloured red. H_2_O molecules are shown as balls (oxygen, red; hydrogen, grey) and sticks. The docking score (S) for the complex, and the chemical structure of the small molecule, are also provided.

Having selected four potential inhibitor target sites within EC-LigA, we next wanted to screen for small molecules predicted to bind at one or more of these sites. We used *in silico* molecular docking to dock 800 commercially available small molecules from an Acros-Organics screening library, which included hydrocarbon, polycyclic and aromatic small molecules, at each of the potential inhibitor target sites. Docking was performed in MOE with the potential inhibitor target site as a rigid body and the small molecules allowed conformational flexibility. Small molecules were ranked from lowest energy/docking score to highest energy/docking score for each potential inhibitor target site. The lowest energy conformation of the EC-LigA-small molecule complex for the top two small molecules for each potential inhibitor target site is shown in Figure 3, together with the corresponding docking score (S) and the chemical structure of the small molecule. 

The two small molecules that docked into Site 1 with the lowest energy were geneticin (also known as G418, but will be referred to as geneticin here) and chlorhexidine. Geneticin docked on the surface of the OB-fold domain in a conformation that was exposed to solvent. Interactions were predicted between geneticin and EC-LigA residues R325, D326, D398 and R400. Chlorhexidine was similarly predicted to interact with EC-LigA D326 and D398. Glutathione and imidazolidinyl urea both docked into Site 2. Glutathione docked in a conformation that was solvent exposed and interactions were predicted with EC-LigA K457, I458, D460, K465 and E486. Imidazolidinyl urea was predicted to interact with EC-LigA G453, K457, E486, essential amino acid R487 and M488. The small molecules that docked with the lowest energy into Site 3 were 5-azacytidine and imidazo[1,2-a]pyridine-7-carboxylic acid (imidazo[1,2-a]pyridine-7-C). Both 5-azacytidine and imidazo[1,2-a]pyridine-7-C were predicted to interact with the essential amino acid EC-LigA K115 which is the EC-LigA adenylation site. 5-azacytidine was also predicted to interact with Q113 and L116 (the x in the KxDG motif) while imidazo[1,2-a]pyridine-7-C was also predicted to interact with the essential amino acid K290 (contacts NAD^+^). Finally, bestatin and 2-(aminomethyl)imidazole docked into Site 4 and were predicted to interact with the essential amino acid D36 which is required for NAD^+^ binding and formation of the enzyme-adenylate in the first step of the ligase reaction. Bestatin was also predicted to interact with EC-LigA R43, A65 and S147 and 2-(aminomethyl)imidazole was also predicted to interact with A68, F70, S147 and L210 which is proximal to essential amino acid R208 (DNA binding).

### 2.3. In Vitro Screening of Candidate EC-LigA Small Molecule Inhibitors

**Figure 4:**
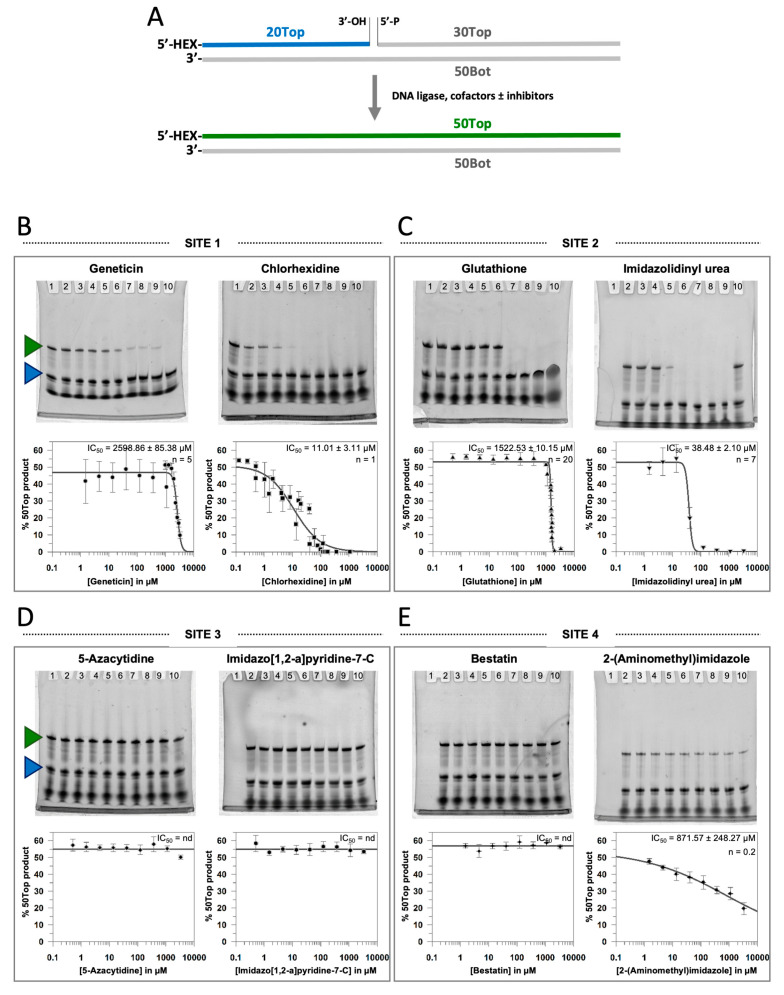
*In vitro* screening of candidate EC-LigA small molecule inhibitors. (**A**) A schematic of the *in vitro* ligation assay. A 50 bp nicked DNA substrate was prepared by annealing a 5′ hexachlorofluorescein (HEX) labelled, 3′ hydroxyl 20 nt single stranded oligonucleotide (20Top, blue) and an unlabelled, 5′ phosphate 30 nt single stranded oligonucleotide (30Top) to a complementary unlabelled 50 nt single stranded oligonucleotide (50Bot). Subsequent ligation of 20Top and 30Top, by EC-LigA produced a 50 bp double stranded DNA product comprising 5′ HEX labelled 50 nt DNA (50Top, green) and the complementary unlabelled 50 nt DNA 50Bot. (**B**) Representative 10% (*w*/*v*) denaturing PAGE of *in vitro* ligase assays performed with 120 nM nicked DNA substrate, 30 nM EC-LigA and 26 µM NAD^+^ in reaction buffer (30 mM Tris HCl, pH 8.0, 4 mM MgCl_2_, 1 mM DTT, and 50 µg/mL BSA) in the presence of increasing concentrations of geneticin (0–3333 µM) or chlorhexidine (0–3333 µM) at room temperature for 5 min. Blue and green triangles mark the HEX labelled 20Top (substrate) and 50Top (product) DNA species, respectively. Gels were quantitated using ImageJ and the mean percentage 50Top DNA product was plotted against small molecule concentration. Error bars represent the standard error of the mean for a minimum of two experimental repeats. All data points were simultaneously fitted to a three-parameter IC_50_ inhibition function (black line) to determine the IC_50_ and *n*, which are reported in the top right corner of the plots. (**C**) As for (**B**) but for increasing concentrations of glutathione (0–3333 µM) or imidazolidinyl urea (0–3333 µM). (**D**) As for (**B**) but for increasing concentrations of 5-azacytidine (0–3333 µM) or imidazo[1,2-a]pyridine-7-C (0–3333 µM). (**E**) As for (**B**) but for increasing concentrations of bestatin (0–3333 µM) or 2-(aminomethyl)imidazole (0–3333 µM).

In order to determine whether the small molecules that docked into the potential inhibitor target sites in EC-LigA are able to inhibit EC-LigA, we decided to screen them using an *in vitro* ligase assay. A 50 bp nicked DNA substrate was designed and prepared by annealing a 5′ hexachlorofluorescein (HEX) labelled, 3′ hydroxyl 20 nt single stranded oligonucleotide (20Top) and an unlabelled, 5′ phosphate 30 nt single stranded oligonucleotide (30Top) to a complementary unlabelled 50 nt single stranded oligonucleotide (50Bot) (Figure 4A). Incubation of this nicked DNA substrate with EC-LigA in the presence of NAD^+^ cofactor should lead to phosphodiester bond formation between the 3′ hydroxyl of 20Top and the 5′ phosphate of 30Top to produce a 50 bp double stranded DNA product. The ligation reaction can be evaluated by resolving the 50 nt 5′ HEX labelled single stranded DNA and the 20 nt 5′ HEX labelled 20Top single stranded DNA by denaturing polyacrylamide gel electrophoresis (PAGE). The inhibitory activity of small molecules on EC-LigA activity can then be assessed by comparing the extent of ligation (the percentage of 50 nt 5′ HEX labelled single stranded DNA produced) in the absence of small molecule to the extent of ligation in the presence of small molecule. Small molecule inhibitors of EC-LigA will reduce the percentage of 50 nt 5′ HEX labelled single stranded DNA produced.

The inhibitory activity of each of the top two small molecules predicted to bind to each of the potential inhibitor target sites within EC-LigA (Figure 3), eight small molecules in total, was evaluated using the *in vitro* ligase assay. In the absence of small molecule, under the experimental conditions tested, approximately 50% ligation occurred (Figure 4B–E). The extent of ligation was then determined in the presence of a range of concentrations for each of the small molecules. For three of the small molecules (5-azacytidine, imidazo[1,2-a]pyridine-7-C and bestatin), the extent of ligation was approximately 50% at all of the small molecule concentrations tested indicating that these small molecules do not inhibit EC-LigA under these experimental conditions. The remaining five small molecules (geneticin, chlorhexidine, glutathione, imidazolidinyl urea and 2-(aminomethyl)imidazole) all inhibited the ligation reaction in a dose-dependent manner. Complete inhibition of ligation was observed for geneticin, chlorhexidine, glutathione and imidazolidinyl urea. In contrast, 2-(aminomethyl)imidazole appeared to dramatically affect ligase activity at low concentrations, but this inhibition was only partial and complete inhibition was not observed, even at the highest concentration tested (3333 µM).

In order to compare the relative potency of the identified small molecule inhibitors, we decided to determine the concentration of each one that results in a 50% reduction in ligase activity (the apparent half maximal inhibitory concentration (IC_50_)). This is a common approach for comparing the activity of inhibitors during the inhibitor screening process. Dose-response curves were fitted with a three-parameter inhibition curve (see Materials and Methods) to determine the IC_50_ and the Hill slope factor (*n*) for each of the inhibitory small molecules (Figure 4B–E). The apparent IC_50_ for 2-(aminomethyl)imidazole is likely an overestimation due to the partial inhibition observed with this small molecule, but it has been included to allow comparison with the other data. The IC_50_ for geneticin, glutathione and 2-(aminomethyl)imidazole were in the low mM range (IC_50_ = 2.59, 1.52 and 0.87 mM, respectively) consistent with weak inhibition of EC-LigA. Chlorhexidine and imidazolidinyl urea are more potent inhibitors of EC-LigA with an IC_50_ of 11.0 µM and 38.5 µM, respectively. The Hill slope factor (*n*), a measure of how responsive the enzyme is to changes in inhibitor concentration, also varied for each of the small molecule inhibitors (Figure 4B–E; Figure S2). The slope factor for chlorhexidine was 1 which is expected for a standard inhibitory response. Geneticin, glutathione and imidazolidinyl urea all had slope factors > 1 (*n* = 5, 20 and 7, respectively) indicating that the enzyme is responsive to small changes in concentration of these small molecules over a narrow concentration range. In contrast, 2-(aminomethyl)imidazole had a slope factor < 1 (*n* = 0.2) indicating that EC-LigA is relatively insensitive to small changes in concentration of this small molecule over a broad concentration range. This might be expected if inhibition is nonspecific.

## 3. Discussion

DNA ligases are an attractive antibacterial target due to significant differences between bacterial NAD^+^ ligases and eukaryotic ATP ligases. Antibacterial targeting of bacterial NAD^+^ ligases has focused on the NAD^+^ binding Ia domain that is unique to the bacterial enzymes and/or NAD^+^ analogues [14,27,28,29,30,31]. In this study, we aimed to investigate the potential for targeting other regions of the *E. coli* NAD^+^ ligase, LigA, using a global *in silico* molecular docking approach. Four potential inhibitor target sites were identified in distinct regions of the enzyme (Site 1, OB-fold domain; Site 2, HhH domain; Site 3, NTase domain; Site 4, Ia/NTase domains). A screening library of 800 small molecules was docked into each of these sites and eight small molecules (two targeting each of the sites) were selected for *in vitro* inhibitor screening. Of these eight small molecules, five inhibited EC-LigA *in vitro*, collectively targeting three out of four of the target sites. A summary of the inhibitory properties of these five small molecules is presented in Table 1.

### 3.1. Novel EC-LigA Small Molecule Inhibitors

Since DNA ligases are essential [3,4,5,6], specific inhibitors of bacterial NAD^+^ ligases have the potential to have antibacterial activity. Three of the small molecule EC-LigA inhibitors identified in this study (geneticin, chlorhexidine and imidazolidinyl urea) are known to have antimicrobial properties. Geneticin is a gentamicin-family aminoglycoside antibiotic that is known to block polypeptide synthesis and ribosome translation by binding the 16S ribosomal RNA subunit [40,41,42]. Consequently, geneticin is unlikely to be of use as an antimicrobial targeting DNA ligase. However, the basis of the antimicrobial activity of chlorhexidine, which is used in many medical and dental applications as a topical disinfectant, including in the coatings on medical devices and as an antiseptic in oral and skin treatments [43,44], and imidazolidinyl urea, which is an additive used in cosmetics [45], is currently unknown. It is possible that DNA ligase may be a target for these compounds *in vivo*, provided they pass through the bacterial membrane. A fourth inhibitor, glutathione, is a metabolic tripeptide involved in regulating cellular redox state and detection of reactive-oxygen species in *E. coli* [46,47]. There is growing evidence that enzymes involved in nucleic acid metabolism are regulated/inhibited by metabolites [33,48,49]. It would be interesting to investigate if this is also the case for DNA ligases.

The relatively modest IC_50_ for chlorhexidine and imidazolidinyl urea (≈11–38 µM) and the relatively high IC_50_ values for the other three novel inhibitors (Table 1), suggests that these small molecules might not be considered as lead molecules *per se*, but might serve as chemical building blocks for improved inhibitors in the future development of antibacterial strategies targeting EC-LigA, rather than ready-to-go antibacterials. However, even if the potential of these specific small molecule inhibitors is limited as antibacterials, they could still be of value as research tools to investigate ligase mechanism and function. 

### 3.2. Novel Inhibitor Target Sites within EC-LigA

Four sites were selected as potential inhibitor target sites. Three of the four sites contained amino acids known to be essential (Site 2/HhH domain; Site 3/NTase domain and Site 4/Ia and NTase domain). Surprisingly, neither of the small molecules that were predicted to bind at Site 3, the site containing the most essential amino acids, inhibited EC-LigA. However, there were no specific interactions predicted between these small molecules and the essential amino acids present in the binding site, although they were predicted to bind to essential amino acids proximal to the site. Imidazolidinyl urea is predicted to bind at Site 2 and interact with R487 that is essential for DNA binding which could explain its inhibitory activity. Similarly, the inhibitory activity of 2-(aminomethyl)imidazole could be explained from the molecular docking. 2-(aminomethyl)imidazole is predicted to bind at Site 4 and interact with D36 of the Ia domain which is essential for NAD^+^ binding. Of most interest was that, despite a lack of known essential amino acids comprising Site 1 within the OB-fold domain, both of the small molecules that were predicted to bind to this site (geneticin and chlorhexidine) had inhibitory activity against EC-LigA *in vitro*. This suggests that this study may have identified a region of the OB-fold domain, that has not been directly investigated previously, which may be critical for EC-LigA activity. It is possible that small molecule binding to this region of the OB-fold domain could block the conformational changes required of this domain in forming the clamp structure around the nick site. Further investigation, such as site-directed mutagenesis, kinetics or structural studies, will be required to determine the mode of inhibition for each of the identified inhibitory compounds, including whether they do in fact bind at their predicted target sites. Nevertheless, the identified small molecule accessible sites could be promising inhibitor target sites within EC-LigA.

### 3.3. Approach

The strategy of combining structure-based *in silico* screening with *in vitro* validation provides a high-throughput method for identifying small molecule inhibitors, provided that structural and functional characterisation of the target protein is available. Previously, we have successfully applied this approach to identify inhibitors of RNase E [32,33]. We have now applied the strategy to identify novel inhibitors of EC-LigA and potentially novel inhibitor target sites within EC-LigA. Although further characterisation of the inhibitors will be required to realise their potential as either antibacterial leads or tool molecules, the hit-rate of 63% (5 out of 8 small molecules screened *in vitro*) demonstrates the power of this approach and we anticipate that this strategy may be of value in targeting NAD^+^ ligases as antibacterial strategy in the future.

## 4. Materials and Methods

### 4.1. Identification of Potential Inhibitor Target Sites within EC-LigA

Molecular Operating Environment (MOE, Chemical Computing Group) molecular docking software [39] was used to prepare an apo-EC-LigA structure. The bound nicked DNA-adenylated and three nonspecific sulphate ions were removed from the 2OWO [16] crystal structure. A protonated and energy minimised apo-EC-LigA was prepared using MOE. The MOE site-finder tool was used to identify solvent-exposed sites with a volume > 5 Å^3^ within the apo-EC-LigA structure. Thirty-two sites were identified and four were selected as potential inhibitor target sites (Figure 2; Tables S2–S5). Small molecule accessible sites were visualised in PyMOL (Schrodinger Inc.). The functional significance of these four sites was assessed by comparing their amino acid composition and structural location to a series of mutational-mapping studies conducted by the Shuman group [34,35,36,37,38] (Table S1; Figure S1) in PyMOL.

### 4.2. In Silico Molecular Docking of Small Molecules into the Potential Inhibitor Target Sites within EC-LigA

Structures for a screening library of 800 commercially available small molecules (Arcos-Organics Inc.) were retrieved from the ZINC database [50] and prepared for docking in MOE. The MOE Dock function was used to dock each of the compounds into each of the potential inhibitor target sites in the apo-EC-LigA structure. The potential inhibitor target site within the apo-EC-LigA structure was held as a rigid body and the small molecules were allowed conformational flexibility. “Triangle Matcher” placement methodology and 100 placement poses were used. Apo-EC-LigA-small molecule complexes were scored according to the London dG scoring function to give a docking score (S). The 20 lowest energy/docking score apo-EC-LigA-small molecule complex conformations were retained for each small molecule docked at each potential inhibitor target site. Small molecules were ranked from lowest energy/docking score to highest energy/docking score for each potential inhibitor target site. The top two small molecules for each potential inhibitor target site were selected for screening of their ligase inhibitor activity *in vitro*.

### 4.3. Reagents

Geneticin, chlorhexidine, glutathione disulphide, imidazolidinyl urea, 5-azacytidine, imidazo[1,2-a]pyridine-7-C, bestatin (Ubenimex) and 2-(aminomethyl)imidazole were purchased from Sigma-Aldrich as solids and each was resuspended at a stock concentration of 50 mM in nuclease-free water. Their chemical structures are shown in Figure 3. Oligonucleotides, 5′-HEX-ATCTCGCGTATGGGCCTTCG-OH-3′ (20Top), 5′-p-CTGCTCACAGGACACCTGGTATACGTAATG-OH-3′ (30Top) and 5′-CATTACGTATACCAGGTGTCCTGTGAGCAGCGAAGGCCCATACGCGAGAT (50Bot) were synthesised at 0.2 µmol scale and PAGE-purified by Invitrogen. Each was resuspended at 50 µM in nuclease-free water. EC-LigA (5.4 µM stock, determined by SDS-PAGE) and ligase buffers were from NEB.

### 4.4. Preparation of the 50 bp Nicked DNA Substrate

A mixture of 10 µM 20Top, 10 µM 30Top and 10 µM 50Bot DNAs was prepared in annealing buffer (10 mM Tris-HCl, pH 8.0, 0.4 mM MgCl_2_ and 0.1 mM DTT). The mixture was heated to 95 °C in a thermocycler and allowed to cool to 4 °C at a rate of 0.125 °C per min.

### 4.5. In Vitro Ligase Assays

Ligase reactions (50 µl) containing 120 nM 50 bp nicked DNA substrate, 30 nM EC-LigA and 26 µM NAD^+^ in reaction buffer (30 mM Tris HCl, pH 8.0, 4 mM MgCl_2_, 1 mM DTT, and 50 µg/mL BSA) were incubated at room temperature for 5 min in the absence of small molecule or in the presence of geneticin (1.52, 4.57, 13.72, 41.15, 123.46, 370.37, 1000.00, 1111.11, 1333.33, 1666.67, 2000.00, 2333.33, 2666.67, 3000.00 or 3333.33 µM), chlorhexidine (0.13, 0.26, 0.51, 0.52, 1.04, 1.52, 2.08, 4.17, 4.57, 8.34, 13.72, 16.67, 20.00, 40.00, 41.15, 60.00, 80.00, 100.00, 120.00, 123.46, 140.00, 160.00, 180.00, 370.37, 1111.11 or 3333.33 µM), glutathione (0.51, 1.52, 4.57, 13.72, 41.15, 123.46, 370.37, 1000.00, 1111.11, 1125.00, 1250.00, 1333.33, 1375.00, 1450.00 1475.00, 1500.00, 1525.00, 1550.00, 1575.00, 1600.00, 1625.00, 1650.00, 1666.67, 1750.00, 1875.00, 2000.00, 2333.33, 2666.67, 3000.00 or 3333.33 µM), imidazolidinyl urea (1.52, 4.57, 13.72, 41.15, 123.46, 370.37, 1111.11 or 3333.33 µM), 5-azacytidine (0.51, 1.52, 4.57, 13.72, 41.15, 123.46, 370.37, 1111.11 or 3333.33 µM), imidazo[1,2-a]pyridine-7-C (0.51, 1.52, 4.57, 13.72, 41.15, 123.46, 370.37, 1111.11 or 3333.33 µM), bestatin (1.52, 4.57, 13.72, 41.15, 123.46, 370.37, 1111.11 or 3333.33 µM)or 2-(aminomethyl)imidazole (1.52, 4.57, 13.72, 41.15, 123.46, 370.37, 1111.11 or 3333.33 µM). Reactions were quenched by the addition of one volume of ligase-stop buffer (10 mM EDTA, pH 8.0, 2 mM NaOH, 80% (*v*/*v*) formamide and 0.1% (*w*/*v*) bromophenol blue).

### 4.6. PAGE Analysis of Ligase Assays

Quenched reactions were heat-denatured at 100°C for 3 min and placed on ice. Reaction products were resolved by 10% (*w*/*v*) denaturing PAGE (10% (*w*/*v*) polyacrylamide gels containing 20% formamide and 7 M urea). Inclusion of formamide in the polyacrylamide gels aided maintenance of single-stranded species during electrophoresis. Gels were visualised using a fluorescence-imager (Fuji FLA-5000) using an excitation wavelength of 532 nm. Digitised images were quantitated using ImageJ software (NIH) and the percentage of 20Top (substrate) and 50Top (product) in each lane was calculated. Data were fit to a three-parameter IC_50_ inhibition function:

$$y=\frac{Y_{max}}{1+{\left(\frac{x}{IC_{50}}\right)}^{n}}$$

In this equation, *y* is the percentage of 50Top (product) at small molecule inhibitor concentration *x*; *Y_max_* is the theoretical maximal percentage of 50Top (product) that can be formed, *IC_50_* is the concentration of small molecule inhibitor at half *Y_max_*, and *n* is the Hill slope factor.

## 5. Conclusions

Most NAD^+^ bacterial ligase inhibitors to date have been heterocycles such as pyridochromanones, pyrimido compounds, NAD^+^- or adenine-based derivatives [14,27,28,29,30,31]. These most likely compete with NAD^+^ for binding to domain Ia, or block AMP transfer to the NTase domain. Our approach has been to explore other small molecule accessible pockets within EC-LigA with the aim of finding new lead compound inhibitors and/or inhibitor target sites that could be pursued either as an antibacterial strategy or that could be useful research tools for investigating the three-step ligase reaction mechanism. We have identified one potentially novel inhibitor target site within EC-LigA and five new inhibitor candidates for further study.

## Figures and Tables

**Table 1:** A summary of the inhibitory properties of the five small molecule inhibitors of EC-LigA.

Small Molecule Inhibitor	Target Site/Domain	Predicted Interactions with Essential Amino Acids	Docking Score (kcal/mol)	IC_50_ (µM)	Hill Slope Factor (*n*)
Geneticin	Site 1/OB-fold	-	−5.35	2598.86 ± 85.38	5
Chlorhexidine	Site 1/OB-fold	-	−4.88	11.01 ± 3.11	1
Glutathione	Site 2/HhH	-	−6.27	1522.53 ± 10.15	20
Imidazolidinyl urea	Site 2/HhH	R487	−5.18	38.48 ± 2.10	7
2-(aminomethyl) imidazole	Site 4/Ia-NTase	D36	−3.85	871.57 ± 248.27	0.2

## Data Availability

The data presented in this study are available on request from the corresponding author.

## Supplementary Materials

The following are available online. Table S1: A summary of mutation-mapping studies of EC-LigA performed by the Shuman group, Table S2: Properties of potential inhibitor target Site 1, Table S3: Properties of potential inhibitor target Site 2, Table S4: Properties of potential inhibitor target Site 3, Table S5: Properties of potential inhibitor target Site 4, Figure S1: Evaluation of the functional significance of potential inhibitor target sites, Figure S2: Comparison of the inhibitory activity of small molecules targeting EC-LigA.

## Sample Availability

Not available.
