# Supplementary material for: Identification of Novel Inhibitors of Escherichia coli DNA Ligase (LigA)
Source: Molecules. 2021 Apr 25;26(9):2508. doi: 10.3390/molecules26092508 (PMC8123306; doi:10.3390/molecules26092508)
Supplement: Supplementary file 1 [file molecules-26-02508-s001.zip › molecules-1179287-supplementary.pdf]

Supplementary Materials

Identification of Novel Inhibitors of *Escherichia coli* DNA Ligase (LigA)

Arqam Alomari[1,2†], Robert Gowland[1†], Callum Southwood[1†], Jak Barrow[1], Zoe Bentley[1], Jashel Calvin-Nelson[1], Alice Kaminski[1], Matthew LeFevre[1], Anastasia J. Callaghan[1], Helen A. Vincent[1] and Darren M. Gowers[1*].

[1] School of Biological Sciences and Institute of Biological and Biomedical Sciences, University of Portsmouth, Portsmouth, PO1 2DY, United Kingdom.

[2] Current address: Department of Basic Sciences, College of Agriculture and Forestry, University of Mosul, Mosul, 41002, Iraq.

[†] These authors contributed equally to this work.

* Correspondence: darren.gowers@port.ac.uk

Supplementary References

1. Sriskanda, V., Schwer, B., Ho, C.K. and Shuman, S. Mutational analysis of *Escherichia coli* DNA ligase identifies amino acids required for nick-ligation *in vitro* and for *in vivo* complementation of the growth of yeast cells deleted for CDC9 and LIG4. *Nucleic Acids Res.* **27**, 3953-3963. doi: 10.1093/nar/27.20.3953.

2. Sriskanda, V. and Shuman, S. (2002) Conserved residues in domain Ia are required for the reaction of *Escherichia coli* DNA Ligase with NAD+. *J. Biol. Chem.* **277**, 9695-9700. doi: 10.1074/jbc.M111164200.

3. Zhu, H. and Shuman, S. (2005) Structure-guided mutational analysis of the nucleotidyltransferase domain of *Escherichia coli* NAD+-dependent DNA ligase (LigA). *J. Biol. Chem.* **280**, 12137–12144. doi: 10.1074/jbc.M413685200.

4. Wang, L.K., Nair, P.A. and Shuman, S. (2008) Structure-guided mutational analysis of the OB, HhH, and BRCT domains of *Escherichia coli* DNA ligase. *J Biol Chem*. 2008 **283**, 23343-23352. doi: 10.1074/jbc.M802945200.

5. Wang, L.K., Zhu, H. and Shuman, S. (2009) Structure-guided mutational analysis of the nucleotidyltransferase domain of *Escherichia coli* DNA Ligase (LigA). *J. Biol. Chem.* **284**, 8486–8494. doi: 10.1074/jbc.M808476200.

6. Nandakumar, J., Nair, P.A. and Shuman, S. (2007) Last stop on the road to repair: structure of *E. coli* DNA ligase bound to nicked DNA-adenylate. *Mol. Cell*, **26**, 257-271. doi: 10.1016/j.molcel.2007.02.026.

Supplementary Table S1. A summary of mutation-mapping studies of EC-LigA performed by the Shuman group [1-5]. Amino acids are considered essential for EC-LigA function if they result in ≥90% inhibition of ligase activity when mutated.

Residue	Domain	Role	Mutation	% Ligase Inhibition	Reference
D285	NTase	Metal-binding at active site	A	>99.99	Sriskanda *et al*., 1999 [1]
Y22	Ia	NAD+ binding	A	>99.9	Sriskanda and Shuman, 2002 [2]
Y35	Ia	NAD+ binding	S	>99.9	Sriskanda and Shuman, 2002 [2]
K115	NTase	Adenylated residue of KxDG motif	A	>99.9	Sriskanda *et al*., 1999 [1]
K115	NTase	Adenylated residue of KxDG motif	R	>99.9	Zhu and Shuman, 2005 [3]
K115	NTase	Adenylated residue of KxDG motif	Q	>99.9	Zhu and Shuman, 2005 [3]
D117	NTase	KxDG motif	A	>99.9	Sriskanda *et al*., 1999 [1]
D117	NTase	KxDG motif	N	>99.9	Zhu and Shuman, 2005 [3]
G118	NTase	KxDG motif	A	>99.9	Zhu and Shuman, 2005 [3]
R136	NTase	Coordinates reactive phosphates, needed for steps 1 and 2	A	>99.9	Wang, Zhu and Shuman, 2009 [5]
R136	NTase	Coordinates reactive phosphates, needed for steps 1 and 2	Q	>99.9	Wang, Zhu and Shuman, 2009 [5]
E173	NTase	Contacts NAD+	A	>99.9	Zhu and Shuman, 2005 [3]
E173	NTase	Contacts NAD+	D	>99.9	Zhu and Shuman, 2005 [3]
E173	NTase	Contacts NAD+	Q	>99.9	Zhu and Shuman, 2005 [3]
R200	NTase	Ia/Ntase interface, DNA binding	A	>99.9	Zhu and Shuman, 2005 [3]
R200	NTase	Ia/Ntase interface, DNA binding	K	>99.9	Zhu and Shuman, 2005 [3]
R200	NTase	Ia/Ntase interface, DNA binding	Q	>99.9	Zhu and Shuman, 2005 [3]
R208	NTase	Ia/Ntase interface, DNA binding	Q	>99.9	Zhu and Shuman, 2005 [3]
D285	NTase		N	>99.9	Zhu and Shuman, 2005 [3]
K314	NTase	Contacts NAD+	A	>99.9	Sriskanda *et al*., 1999 [1]
K314	NTase	Contacts NAD+	Q	>99.9	Zhu and Shuman, 2005 [3]
C408	Zn	Metal-binding	A	>99.9	Sriskanda *et al*., 1999 [1]
C411	Zn	Metal-binding	A	>99.9	Sriskanda *et al*., 1999 [1]
C432	Zn	Metal-binding	A	>99.9	Sriskanda *et al*., 1999 [1]
Y22	Ia	NAD+ binding	S	99.8	Sriskanda and Shuman, 2002 [2]
D32	Ia	NAD+ binding	A	99.8	Sriskanda and Shuman, 2002 [2]
D36	Ia	NAD+ binding	A	99.8	Sriskanda and Shuman, 2002 [2]
R136	NTase	Coordinates reactive phosphates, needed for steps 1 and 2	K	99.8	Wang, Zhu and Shuman, 2009 [5]
D32	Ia	NAD+ binding	E	99.6	Sriskanda and Shuman, 2002 [2]
R379	OB	Stabilises OB fold	A	99.3	Wang, Nair and Shuman, 2008 [4]
R333	OB	DNA binding	A	99.2	Wang, Nair and Shuman, 2008 [4]
G489	HhH	DNA binding	D	99.2	Wang, Nair and Shuman, 2008 [4]
G521	HhH	DNA binding	V	99.1	Wang, Nair and Shuman, 2008 [4]
G553	HhH	DNA binding	D	99.1	Wang, Nair and Shuman, 2008 [4]
R171	NTase	Stabilise active domain fold	A	>99	Wang, Nair and Shuman, 2008 [4]
G455	HhH	DNA binding	V	>99	Wang, Nair and Shuman, 2008 [4]
G455	HhH	DNA binding	D	>99	Wang, Nair and Shuman, 2008 [4]
R277	NTase	Stabilises NTase active site fold	A	98.9	Zhu and Shuman, 2005 [3]
R208	NTase	Ia/Ntase interface, DNA binding	K	98.8	Zhu and Shuman, 2005 [3]
R379	OB	Stabilises OB fold	Q	98.8	Wang, Nair and Shuman, 2008 [4]
R277	NTase	Stabilises NTase active site fold	Q	98.2	Zhu and Shuman, 2005 [3]
Y35	Ia	NAD+ binding	A	98	Sriskanda and Shuman, 2002 [2]
R208	NTase	Ia/Ntase interface, DNA binding	A	98	Zhu and Shuman, 2005 [3]
R218	NTase	Bind phophodiester bond flanking nick	A	98	Wang, Zhu and Shuman, 2009 [5]
G521	HhH	DNA binding	A	98	Wang, Nair and Shuman, 2008 [4]
G521	HhH	DNA binding	D	97.4	Wang, Nair and Shuman, 2008 [4]
R614	BRCT		A	97.4	Wang, Nair and Shuman, 2008 [4]
D117	NTase	KxDG motif	E	97	Zhu and Shuman, 2005 [3]
R171	NTase	Stabilise active domain fold	Q	97	Wang, Zhu and Shuman, 2009 [5]
K290	NTase	Contacts NAD+/contacts AMP adenine	Q	97	Wang, Zhu and Shuman, 2009 [5]
G489	HhH	DNA binding	V	96.8	Wang, Nair and Shuman, 2008 [4]
D36	Ia	NAD+ binding	E	96	Sriskanda and Shuman, 2002 [2]
R218	NTase	Bind phophodiester bond flanking nick	Q	96	Wang, Zhu and Shuman, 2009 [5]
D285	NTase		E	96	Zhu and Shuman, 2005 [3]
R308	NTase	Bind phophodiester bond flanking nick	Q	96	Wang, Zhu and Shuman, 2009 [5]
V383	OB	Distorts DNA at nick site	A	96	Wang, Nair and Shuman, 2008 [4]
I384	OB	Distorts DNA at nick site	A	96	Wang, Nair and Shuman, 2008 [4]
K314	NTase	Contacts NAD+	R	95	Zhu and Shuman, 2005 [3]
R446	HhH	Domain-domain interaction	A	94.6	Wang, Nair and Shuman, 2008 [4]
R277	NTase	Stabilises NTase active site fold	K	94.4	Zhu and Shuman, 2005 [3]
S81	NTase	DNA binding	A	94	Wang, Zhu and Shuman, 2009 [5]
R487	HhH	DNA binding	A	94	Wang, Nair and Shuman, 2008 [4]
R379	OB	Stabilises OB fold	K	93.7	Wang, Nair and Shuman, 2008 [4]
C426	Zn	Metal-binding	A	93	Sriskanda *et al*., 1999 [1]
K290	NTase	Contacts NAD+/contacts AMP adenine	R	92	Wang, Zhu and Shuman, 2009 [5]
Y22	Ia	NAD+ binding	F	91	Sriskanda and Shuman, 2002 [2]
D32	Ia	NAD+ binding	N	91	Sriskanda and Shuman, 2002 [2]
R308	NTase	Bind phophodiester bond flanking nick	A	91	Wang, Zhu and Shuman, 2009 [5]

H23	Ia	NAD+ binding	A	90	Sriskanda and Shuman, 2002 [2]
D36	Ia	NAD+ binding	N	88	Sriskanda and Shuman, 2002 [2]
K290	NTase	Contacts NAD+/contacts AMP adenine	A	87	Zhu and Shuman, 2005 [3]
T524	HhH	DNA binding	A	87	Wang, Nair and Shuman, 2008 [4]
G286	NTase		A	86	Zhu and Shuman, 2005 [3]
R305	NTase		A	86	Wang, Zhu and Shuman, 2009 [5]
K627	BRCT		A	86	Wang, Nair and Shuman, 2008 [4]
R171	NTase	Stabilise active domain fold	K	85	Wang, Zhu and Shuman, 2009 [5]
R97	NTase	Bind phophodiester bond flanking nick	A	83	Wang, Zhu and Shuman, 2009 [5]
R218	NTase	Bind phophodiester bond flanking nick	K	81	Wang, Zhu and Shuman, 2009 [5]
R342	OB		A	80	Wang, Nair and Shuman, 2008 [4]
N84	NTase	DNA binding	A	79	Wang, Zhu and Shuman, 2009 [5]
T334	OB	DNA binding	A	79	Wang, Nair and Shuman, 2008 [4]
R447	HhH	Domain-domain interaction	A	79	Wang, Nair and Shuman, 2008 [4]
T135	NTase	DNA binding	A	78	Wang, Zhu and Shuman, 2009 [5]
T524	HhH	DNA binding	S	78	Wang, Nair and Shuman, 2008 [4]
Y35	Ia	NAD+ binding	F	77	Sriskanda and Shuman, 2002 [2]
Y225	NTase		A	75	Sriskanda et al., 1999 [1]
R487	HhH	DNA binding	K	75	Wang, Nair and Shuman, 2008 [4]
R487	HhH	DNA binding	Q	75	Wang, Nair and Shuman, 2008 [4]
G489	HhH	DNA binding	A	75	Wang, Nair and Shuman, 2008 [4]
N198	NTase		A	74	Zhu and Shuman, 2005 [3]
N201	NTase	DNA binding	A	74	Wang, Zhu and Shuman, 2009 [5]
R101	NTase	Bind phophodiester bond flanking nick	A	72	Wang, Zhu and Shuman, 2009 [5]
R97	NTase	Bind phophodiester bond flanking nick	Q	70	Wang, Zhu and Shuman, 2009 [5]
Q209	NTase		A	67	Wang, Zhu and Shuman, 2009 [5]
N355	OB		A	66.7	Wang, Nair and Shuman, 2008 [4]
G553	HhH	DNA binding	A	65	Wang, Nair and Shuman, 2008 [4]
G172	NTase		A	64	Zhu and Shuman, 2005 [3]
D138	NTase		A	63	Zhu and Shuman, 2005 [3]
K651	BRCT		A	63	Wang, Nair and Shuman, 2008 [4]
Q72	NTase	DNA binding	A	62	Wang, Zhu and Shuman, 2009 [5]
T524	HhH	DNA binding	V	62	Wang, Nair and Shuman, 2008 [4]
G553	HhH	DNA binding	V	62	Wang, Nair and Shuman, 2008 [4]
S206	NTase		A	61	Wang, Zhu and Shuman, 2009 [5]
E113	NTase		A	60	Sriskanda et al., 1999 [1]
K648	BRCT		A	60	Wang, Nair and Shuman, 2008 [4]
G455	HhH	DNA binding	A	56	Wang, Nair and Shuman, 2008 [4]
E143	NTase		A	48	Zhu and Shuman, 2005 [3]
D450	HhH	Domain-domain interaction	A	45	Wang, Nair and Shuman, 2008 [4]
D283	NTase		A	43	Zhu and Shuman, 2005 [3]
R97	NTase	Bind phophodiester bond flanking nick	K	37	Wang, Zhu and Shuman, 2009 [5]
D452	HhH	DNA binding	A	32	Wang, Nair and Shuman, 2008 [4]
E319	NTase		A	31	Sriskanda et al., 1999 [1]
D551	HhH		A	29	Wang, Nair and Shuman, 2008 [4]
V288	NTase		A	24	Zhu and Shuman, 2005 [3]
R308	NTase	Bind phophodiester bond flanking nick	K	20	Wang, Zhu and Shuman, 2009 [5]
Q318	NTase		A	20	Sriskanda et al., 1999 [1]
H23	Ia	NAD+ binding	Y	12	Sriskanda and Shuman, 2002 [2]
E10	Ia		A	10	Sriskanda and Shuman, 2002 [2]
R510	HhH		A	0	Wang, Nair and Shuman, 2008 [4]
L119	NTase		A	-10	Zhu and Shuman, 2005 [3]
E519	HhH		A	-20	Wang, Nair and Shuman, 2008 [4]

Supplementary Table S2. Properties of potential inhibitor target Site 1. Amino acids comprising potential inhibitor target Site 1 (volume = 12 Å^3).

Domain/Residue	Essential	Function	Predicted interaction with geneticin	Predicted interaction with chlorhexidine
OB-fold/R325	Unknown	Unknown	Y	
OB-fold /I371	Unknown	Unknown		
OB-fold /G372	Unknown	Unknown		
OB-fold /D398	Unknown	Unknown	Y	Y
OB-fold /T399	Unknown	Unknown		
OB-fold /R400	Unknown	Unknown	Y	

Supplementary Table S3. Properties of potential inhibitor target Site 2. Amino acids comprising potential inhibitor target Site 2 (volume = 19 Å^3).

**Domain/Residue**	**Essential**	**Function**	**Predicted interaction with glutathione**	**Predicted interaction with imidazolidinyl urea**
HhH/K456	Unknown	Unknown		
HhH/I458	Unknown	Unknown	Y	
HhH/D460	Unknown	Unknown	Y	
HhH/Q461	Unknown	Unknown		
HhH/L485	Unknown	Unknown		
HhH/E486	Unknown	Unknown	Y	Y
**HhH/R487**	**Yes**	**DNA binding**		**Y**
HhH/M488	Unknown	Unknown		Y

Supplementary Table S4. Properties of potential inhibitor target Site 3. Amino acids comprising potential inhibitor target Site 3 (volume = 27 Å^3).

Domain/Residue	Essential	Function	Predicted interaction with 5-azacytidine	Predicted interaction with imidazo[1,2-a]pyridine-7-C
NTase/L116	Unknown	x in KxDG motif	Y	
NTase/D117	Y	KxDG motif		
NTase/G118	Y	KxDG motif		
NTase/E173	Y	Contacts NAD$^+$		
NTase/F175	Unknown	Unknown		
NTase/L176	Unknown	Unknown		
NTase/P177	Unknown	Unknown		
NTase/Q178	Unknown	Unknown		
NTase/P199	Unknown	Unknown		
NTase/R200	Y	DNA binding		
NTase/A203	Unknown	Unknown		
NTase/F282	Unknown	Unknown		
NTase/D283	N	Unknown		

Supplementary Table S5. Properties of potential inhibitor target Site 4. Amino acids comprising potential inhibitor target Site 4 (volume = 41 Å^3).

Domain/Residue	Essential	Function	Predicted interaction with bestatin	Predicted interaction with 2-(aminomethyl)imidazole
Ia/Y35	Y	NAD^+ binding		
**Ia/D36**	**Y**	**NAD^+ binding**	**Y**	**Y**
Ia/A65	Unknown	Unknown	Y	
Ia/P66	Unknown	Unknown		
Ia/L67	Unknown	Unknown		
Ia/A68	Unknown	Unknown		Y
Ia/A69	Unknown	Unknown		
NTase/F70	Unknown	Unknown		Y
NTase/D144	Unknown	Unknown		
NTase/I145	Unknown	Unknown		
NTase/T146	Unknown	Unknown		
NTase/S147	Unknown	Unknown	Y	Y
NTase/N148	Unknown	Unknown		
NTase/Q209	N	Unknown		
NTase/L210	Unknown	Unknown		Y
NTase/D211	Unknown	Unknown		
NTase/P212	Unknown	Unknown		

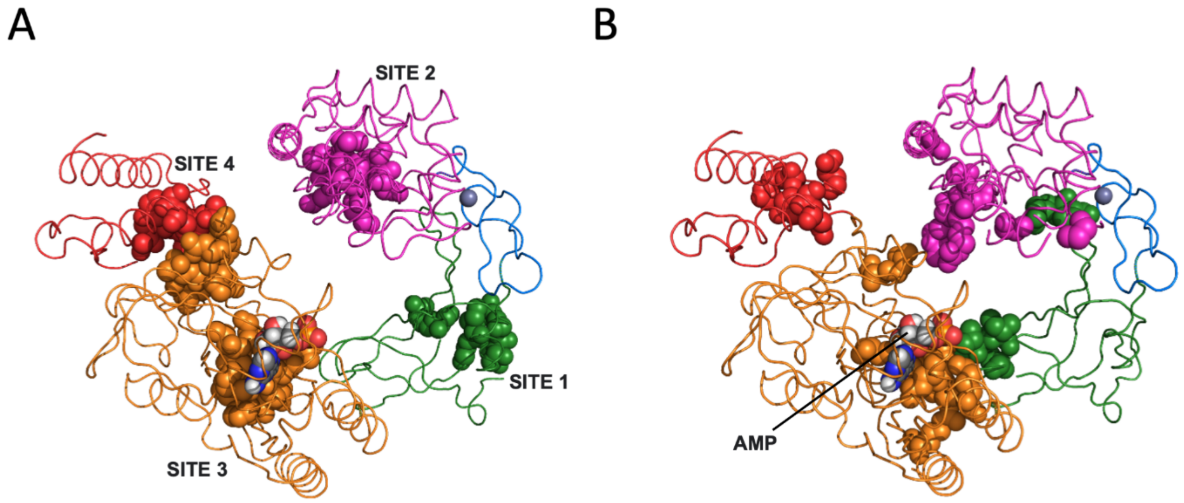

Supplementary Figure S1. Evaluation of the functional significance of potential inhibitor target sites. Structure of EC-LigA (wire) coloured by domain (Ia, red; NTase, orange; OB-fold, green; Zn finger, blue; HhH, magenta) (PDB 2OWO [6] with the nicked DNA removed). The AMP from the nicked DNA-adenylate (grey, red and blue spheres) binds in the AMP-binding pocket within SITE 3. A Zn^{2+} ion (purple sphere) is coordinated by the Zn finger domain. (A) The amino acids forming small molecule accessible sites (spheres, coloured according to domain location) identified using the MOE site-finder tool (as shown in Figure 2). The sites are labelled SITE 1 to SITE 4. (B) The amino acids that are considered to be essential for EC-LigA function based on them resulting in ≥90% inhibition of ligase activity when mutated [1-5] (Supplementary Table S1).

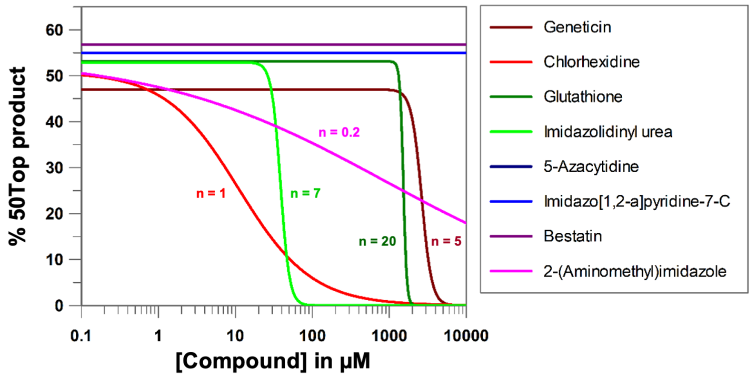

Supplementary Figure S2. Comparison of the inhibitory activity of small molecules targeting EC-LigA. Fits to the data shown in Figure 4B-E plotted as a semi-log plot. Values for the Hill slope factor (n) are indicated.
